# The Role of Mammalian Reservoir Hosts in Tick-Borne Flavivirus Biology

**DOI:** 10.3389/fcimb.2018.00298

**Published:** 2018-08-28

**Authors:** Luwanika Mlera, Marshall E. Bloom

**Affiliations:** Biology of Vector-Borne Viruses Section, Laboratory of Virology, Rocky Mountain Laboratories, National Institute of Allergy and Infectious Diseases (NIAID), National Institutes of Health (NIH), Hamilton, MT, United States

**Keywords:** tick-borne flavivirus, tick-borne encephalitis virus, ixodid ticks, mammalian reservoirs, small-to-medium sized mammals

## Abstract

Small-to-medium sized mammals and large animals are lucrative sources of blood meals for ixodid ticks that transmit life-threatening tick-borne flaviviruses (TBFVs). TBFVs have been isolated from various organs obtained from wild-caught *Myodes* and *Apodemus* species in Europe and Asia. Thus, these rodents are well-established reservoirs of TBFVs. Wild-caught *Peromyscus* species have demonstrated seropositivity against Powassan virus, the only TBFV known to circulate in North America, suggesting that they may play an important role in the biology of the virus in this geographic region. However, virus isolation from *Peromyscus* species is yet to be demonstrated. Wild-caught medium-sized mammals, such as woodchucks (*Marmota monax*) and skunks (*Mephitis mephitis*) have also demonstrated seropositivity against POWV, and virus was isolated from apparently healthy animals. Despite the well-established knowledge that small-to-medium sized animals are TBFV reservoirs, specific molecular biology addressing host-pathogen interactions remains poorly understood. Elucidating these interactions will be critical for gaining insight into the mechanism(s) of viral pathogenesis and/or resistance.

## Introduction

The tick-borne flaviviruses (TBFVs) cause up to 15,000 cases each year in Europe despite the availability of several licensed vaccines (Gritsun et al., 2003; Dobler, 2010; LaSala and Holbrook, 2010; Heinz et al., 2013, 2014). This group of closely related agents is comprised of the tick-borne encephalitis virus sero-complex group (TBEV), Kyasanur Forest disease virus (KFDV), Omsk hemorrhagic fever virus (OHFV), Powassan virus/deer tick virus (POWV/DTV) and the naturally attenuated Langat virus. Humans are accidental hosts and suffer from infection mainly following a tick bite from infected ticks. The pathognomonic features of acute TBFV infection are severe neurological syndromes, which include meningitis and encephalitis, but OHFV and KFDV infections are typically associated with a hemorrhagic fever syndrome and may show encephalitis and/or meningoencephalitis as well. The case fatality rate associated with TBFV infections is varied, but can be up to 40%, depending on the virus (Mandl, 2005).

TBFV particles enclose an 11 kb (+)RNA genome and measure ~60 nm in diameter (Füzik et al., 2018). The genome has a single uninterrupted open reading frame (ORF), which serves as both a template for (–)RNA synthesis as well as the mRNA. Translation of the ORF results in a single polyprotein, which is cleaved by host and viral proteases into 3 structural proteins (C, prM/M, and E) and 7 non-structural proteins (NS1, NS2A, NS2B, NS3, NS4A, NS4B, and NS5). An in-depth review of the functions of the TBFV proteins is available elsewhere (Mlera et al., 2014). Untranslated 5′- and 3′-regions (UTRs) that flank the ORF carry signals for replication, translation, cellular localization, and virion packaging (Bidet and Garcia-Blanco, 2014).

In nature, the TBFVs are maintained in a cycle involving infected hard-bodied (ixodid) ticks and small-to-medium sized mammals from which they obtain blood meals (Deardorff et al., 2013; Mlera et al., 2014). This cycling is particularly noteworthy in that the TBFVs must be well-adapted to replication in both arachnid as well as mammalian host systems. The precise ixodid tick species that are responsible for TBFV transmission differ by geographic region. For example, Powassan virus (POWV) is mainly transmitted by *Ixodes scapularis* and *Ixodes cookei* in the USA, but *Hemaphysalis longicornis* is a vector for the same virus in Asia (Ebel, 2010; Fatmi et al., 2017). The European TBEV subtype is transmitted by *Ixodes ricinus*, whereas *I. persulcatus* transmits the Siberian and Far Eastern TBEV subtypes (Rieille et al., 1920, 2014; Dzhivanyan et al., 1974; Lundkvist et al., 2001; LaSala and Holbrook, 2010).

**Figure 1:**
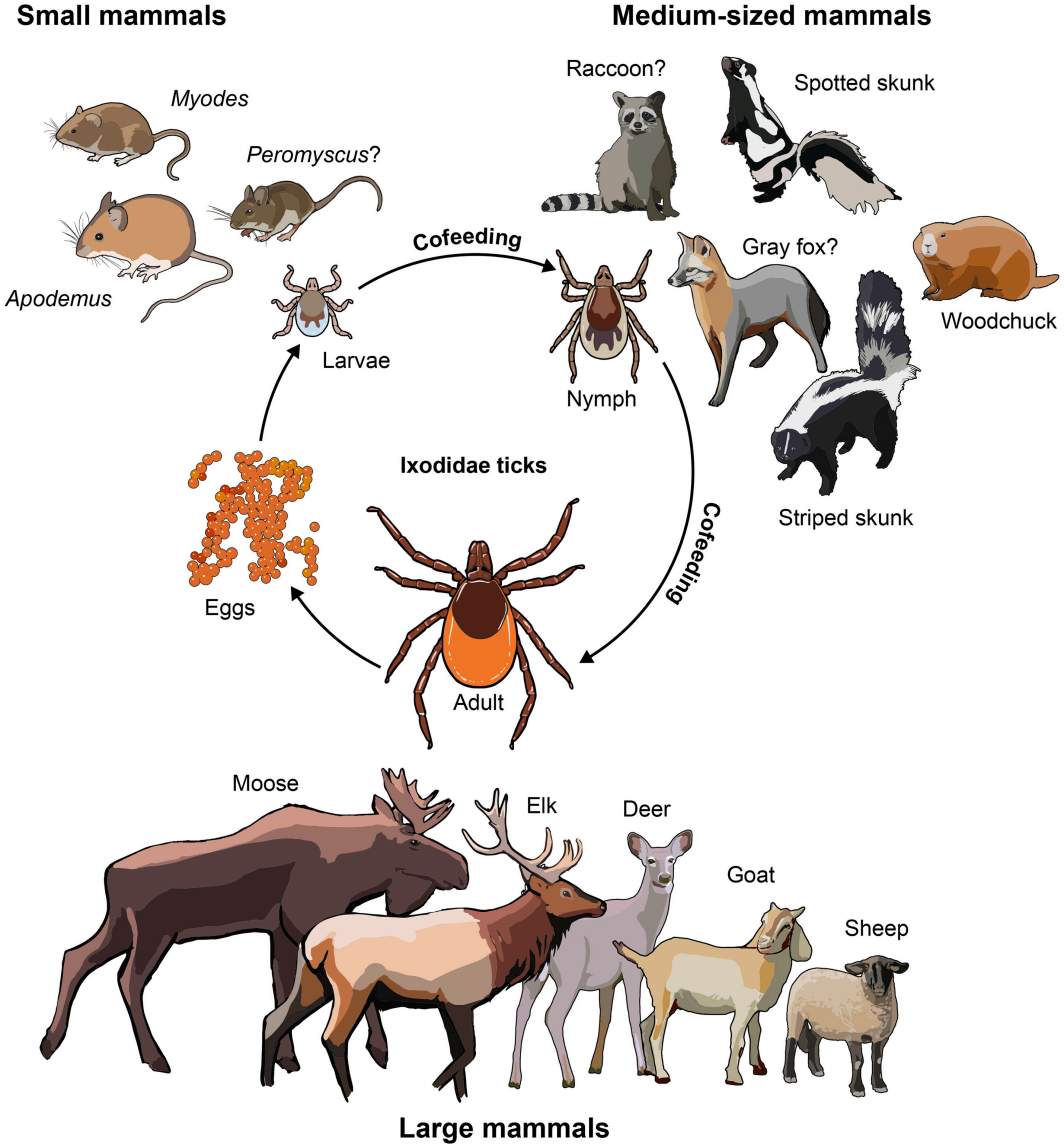
Animals involved in the biology of TBFVs. Small-to-medium sized mammals are known reservoirs of TBFV. Animals with a question mark have been shown to have seroconverted to TBFV infection, but no virus isolation has been demonstrated yet. Large animals play the role of supporting adult tick populations by providing blood meals. Nymphal ticks prefer to feed on medium-sized mammals, whereas larvae favor small rodents. TBFV transmission to naïve ticks is believed to be mostly via co-feeding, and from viremic hosts to a lesser extent (Labuda et al., 1993a, 1997).

The small-to-medium sized mammals that function as unwitting blood banks for ticks may also play a role as TBFV reservoir hosts. Here we define TBFV reservoir hosts as the ecological systems in which the viruses can be indefinitely harbored and from which they may be transmitted to other organisms (Ashford, 2003). This definition would include the ticks, but our focus is on mammalian reservoir hosts. When viremic, these animals can transmit TBFVs to feeding ticks (Khasnatinov et al., 2016). Interestingly, through a process called “co-feeding,” ticks can transmit virus to other ticks when they feed on the same host in close proximity, even if the host is non-viremic (Labuda et al., 1993b,c, 1997; Nuttall and Labuda, 2003; Havlikova et al., 2013). This may be the most important route of transmission from tick-to-tick (Labuda et al., 1993a, 1997). The small-to-medium sized mammals include different rodent species, such as woodchucks (*Marmot monax*), skunks (*Mephitis mephitis*), and squirrels (*Scuiridae*) among others (Figure 1). The role as natural reservoir host is well defined for some of these animals but remains uncertain for others. In this review, we discuss the role played by wild mammalian reservoir hosts of TBFVs in the biology of these increasingly important human pathogens. We hope the review reinvigorates interest and research aimed at understanding this complex host-pathogen relationship.

## TBFV reservoir hosts

Several feral species have been implicated as potential natural hosts of TBFVs. Definitive reservoirs are those from which infectious virus can be isolated, and/or those with high positivity in surveillance studies. Potential reservoir species may have evidence of seropositivity and/or the presence of viral RNA. However, virus may be isolated from spill-over animals not normally considered reservoir hosts.

A general ecological consideration for the rodent reservoir is that they are typically at their peak densities 1 year after a heavy seed crop, usually in the next autumn, with rapid decline over winter (Stenseth et al., 2002). As seeds of various tree species, such as oak and beech are known to provide excellent food for the rodents, it is likely that the rodents' survival is improved for some time following a mast crop (Hansson, 1998). Throughout the next year, they most often remain much below the long-term average and return to “normal levels” as late as on the third year after a heavy seed crop (Hansson, 1971, 1998; Jensen, 1982; Pucek et al., 1993). During each year, the rodent population densities are highest in early winter, followed by a marked decline in spring and a gradual increase in autumn (Gurnell, 1978). These cyclic variations in mammalian reservoir densities may directly impact the tick populations and subsequently the pathogen burden in nature.

In the following sections, we review the animal species that play a major role in the biology of TBFVs and discuss the interactions between host and pathogen.

### TBFVs in *Myodes* species

The *Myodes* genus comprises several species that have a global distribution. The *Myodes glareolus* (bank vole) is a common rodent in Europe and North Asia and its distribution overlaps with regions in which TBEV cases are high (Torre and Arrizabalaga, 2008; Knap et al., 2012). In North America, *M. gapperi* (southern red-backed vole) and *M. rutilus* (northern red-backed vole) are the more common species. The red-backed vole is a major component of the rodent population in deciduous forests of North America (Boonstra and Krebs, 2012). The *M. glareolus* breeding season extends from April to the end of September and the females produce 3–4 litters each (Stenseth et al., 2002). In general, the vole populations are characterized by a marked increase to high populations, followed by a “crash” every 3–4 years (Krebs and Myers, 1974; Ecke et al., 2017).

There is conclusive evidence that the *Myodes* rodents are natural reservoirs for various TBFVs. For example, the TBEV strain CGI223 was isolated from the brain of a *M. glareolus* rodent in 1990 in Záhorská Ves, Slovakia and could be propagated in mouse brains as well as in Vero E6 cells (Frey et al., 2014). TBEV was also isolated from spleen, lung and kidney tissues collected from wild-caught *M. glareolus* which were incidentally coinfected with hantaviruses (Weidmann et al., 2006). The Oshima-C1 TBEV strain is another example of a TBEV isolated from the spleen of wild *M. rufocanus* in Hokkaido, Japan (Takeda et al., 1999). Thus, the *Myodes* species can harbor the TBFVs in different organs.

The percentage of wild-caught *Myodes* rodents with viral RNA in their organs varies from region to region, but rodents from Siberia may be highly infected. The viral loads associated with reservoir host infection may be determined by quantifying genome copy numbers using quantitative PCR. A report from a study done in Siberia showed that 46.2% (18/39) *M. rufocanus* (gray, red-backed vole) were positive for TBEV RNA, whereas 78.1% (25/32) *M. rutilus* had viral RNA in the brain or spleen (Bakhvalova et al., 2016). In some samples, the RNA suggested a mixture of the Siberian (TBEV-Sib) and Far Eastern (TBEV-FE) subtypes (Bakhvalova et al., 2016); this could potentially lead to the generation of recombinant viruses with altered virulence (Bertrand et al., 2012; Fajs et al., 2012; Norberg et al., 2013). A very high TBEV viral load average of 2.5 × 10^9^ RNA copies/mL was reported per organ in *M. glareolus* (Knap et al., 2012), although the levels varied. In this specific case, viral RNA was also detectable in various *M. agrarius* tissues, such as spleen, kidney, lung, liver, heart, blood clots, and brains (Knap et al., 2012). In a separate study, Tonteri and colleagues captured wild rodents in Finland from 2 sites and analyzed the tissue samples for TBEV RNA by PCR. Over a 2-year period, they collected 202 *M. glareolus*, 23 (11.4%) of which were positive for TBEV and in which the RNA was primarily detected in the brain (Tonteri et al., 2011). Almost all the rodents with viral RNA were seropositive for TBEV antibodies (Tonteri et al., 2011). There was no attempt to isolate virus in these studies, but the fact that viral RNA was present in the brain in the absence of clinical signs of disease is a very interesting feature. Viral RNA was also identified in an ecological study in which 4% (6/150) of wild-captured *M. glareolus* rodents were positive for TBEV RNA in the liver (Pintér et al., 2014). The presence of viral RNA in multiple tissues indicates that TBFV infection in the *Myodes* species is not confined to a single organ, and, furthermore, viremia levels suggests that these animals may transmit virus to naïve ticks that feed on them. The high level of viremia, quantified by PCR, is thought to last only for a few days to enable transmission to ticks (Heigl and von Zeipel, 1966; Randolph et al., 1996; Nuttall and Labuda, 2003; Achazi et al., 2011). However, it is noteworthy that the *Myodes* rodents are apparently able to remain asymptomatic while coping with high viral loads in their organs, and this could lead to a sustained virus “leak” into the circulation, hence perpetuating viremia and enabling transmission.

Seroprevalence studies provide additional surrogate evidence for exposure of reservoir hosts to TBFVs, and seropositivity has been demonstrated for the *Myodes* species in several countries. In one study conducted in Switzerland, 3.6% (12/333) wild-captured rodents were seropositive for anti-TBEV antibodies, and 8 of the 12 mice were *M. glareolus* (Burri et al., 2012). In another study conducted in Slovenia, a high prevalence of anti-TBEV antibodies was also observed in *M. agrarius* at 12.5% (39/272) and this seropositivity rate was higher than in other rodent species captured at the same time (Knap et al., 2012). A study in Hungary involving 541 rodents captured over a 4-year period from 2010 to 2014 found 20.5% *M. agrarius* were seropositive against TBEV (Zöldi et al., 2015). Interestingly, in this study, a rate of 0% (0/10) for *M. glareolus* was recorded in 2010, but the incidence increased to 44.4% (8/18) in 2013. However, these incidence rates may be confounded by the small sample sizes. Another interesting observation in this study was the high tick infestation on *M. glareolus*, but not on other rodent species. These differences could be a result of different animal behavior because *Myodes* species move slowly and are more likely to be infested (Zöldi et al., 2015). Higher seropositivity was also associated with older rodents (Zöldi et al., 2015), suggesting repeated exposure and/or a more robust immune response in the adults compared to juveniles.

In North America, *M. rutilus* rodents captured in Central Alaska were 5.8% (14/243) seropositive for POWV/DTV antibodies (Deardorff et al., 2013). *M. gapperi* rodents were captured in Southern Alaska and 6.7% (6/89) of these were positive for antibodies against POWV/DTV (Deardorff et al., 2013). However, this study was limited to serological testing of blood samples only, without virus isolation. Based on the findings that TBEV was readily detectable in *Myodes* species in Europe, attempts to isolate POWV from the same species in the USA should be pursued.

#### Experimental TBFV infection and molecular studies in *Myodes* species

A limited number of reports have described *in vivo* molecular interactions between *Myodes* rodents and TBFVs, or host-pathogen interactions using *Myodes*-derived *in vitro* cell culture models. Such investigations are critical for understanding the basis of TBFV persistence in these reservoir species. Experimental subcutaneous infection of pathogen-free *M. glareolus* with 3 TBEV strains each representing the three subtypes led to viremia by 4 days post infection (dpi) (Tonteri et al., 2013). In these studies, all rodents infected with both the TBEV-Eur and TBEV-FE produced specific IgG antibodies against the virus and had viral RNA in their organs, but only 8/13 rodents that were inoculated with TBEV-Sib tested positive by either method (Tonteri et al., 2013). This showed that there were strain-dependent outcomes in the *Myode*s rodents in the acute phase of the study. A very interesting outcome for the voles was the observation of clinical illness in 2 mice that were inoculated with TBEV-FE. The clinical illness was associated with non-suppurative encephalitis and viral RNA was detected in the brain, spleen and kidney and lung with 1 animal also being viremic. The *Myodes* rodents used in this study were colonized inbred animals and it is not clear if they had acquired any genetic changes that could sponsor susceptibility to severe disease. It is also not known if some rodents that get infected in the wild develop clinical and severe illness, which could lead to their death.

Tonteri and colleagues extended the experimental *Myodes* rodent infection to study viral persistence over a 168-day period. After 109 dpi, viral RNA could only be detected in the brain, an observation which was different from wild-caught *Myodes* which were positive for virus in other organs apart from the brain (Knap et al., 2012; Tonteri et al., 2013). Although viral RNA was detected in the organs of experimentally infected *Myodes* rodents, it is not certain if the RNA was from infectious virus particles because the authors did not attempt to isolate infectious virus. However, virus could be isolated from some, but not all, experimentally infected *Microtus arvalis* rodents, a different vole species, at 100 dpi (Achazi et al., 2011). These differences could be attributed to differences in the way the virus is introduced into the host i.e., tick infection with all the components of tick-saliva vs. needle-inoculation (Hermance and Thangamani, 2015).

One study to examine the response of *Myodes* rodents to TBFVs is that by Stoltz et al. (2011). In this work, the authors experimentally inoculated primary cells obtained from *M. glareolus* fetuses with a human clinical TBEV isolate 1993-783 (Haglund et al., 2003; Stoltz et al., 2011). Inoculation of the *M. glareolus* cells with this TBEV isolate resulted in an infection, which was demonstrated by immunofluorescent staining of viral proteins. The TBEV titer, determined by immunofocus assay, at 12 h post infection (hpi) was surprisingly high at just over 10^6^ ffu/mL, and remained fairly constant as long as 96 hpi (Stoltz et al., 2011). Using qPCR, Stoltz and colleagues further analyzed the expression of *IFN-*β and *MX2*, and reported that *IFN-*β mRNA expression was induced ~100-fold at 12 hpi and remained constant out to 96 hpi. *MX2* mRNA expression rose from a little over 1-fold at 6 and 12 hpi to 100-fold at 24 hpi and peaking at 10,000-fold by 48 hpi. The cells used in this study were a heterogenous population derived from whole-fetus tissues, excluding the head and liver. Thus, the IFN response observed following infection cannot be attributed to a specific cell type. Despite this, it is interesting to note that the cells mounted a strong but ineffective antiviral response, indicating that the IFN response does not necessarily restrict virus infection. Perhaps, the factors used to engage the virus *in vitro* are not as complete as those used *in vivo*.

### Powassan virus in *Peromyscus* species

The *Peromyscus* genus represents the most abundant mixed forest rodent in North America (Bedford and Hoekstra, 2015). The *P. maniculatus* and their congeneric *Peromyscus leucopus* species are mainly distributed in the eastern regions of the USA, coinciding with the geographic regions from which human Powassan virus (POWV) infections have been mostly reported. The optimal habitat for *Peromyscus* mice is the mature woodland with shrubby underwood (Krohne, 1989; Mosheh and O, 2002). Like other rodents, the densities of *Peromyscus* mice are also influenced by seed-crop production as well as weather and habitat changes. For example, the severe ice storm in January 1994 resulted in a decline in *Peromyscus* numbers in northern Illinois from 16.7 individuals per plot to 0.79 individuals (Yunger, 2002). In addition, increased male agonistic behavior is thought to contribute toward poor survival of juvenile *P. maniculatus* during spring and summer (Watts, 1969).

POWV (lineage I) and its close relative DTV (Lineage II) are the only TBFVs known to circulate in North America (Ebel et al., 1999, 2000; Ebel and Kramer, 2004; Ebel, 2010). Some wild-caught mice, such as *P. maniculatus* and *P. truei* were seropositive against POWV/DTV at 6% (2/33) and 22.2% (9/22), respectively (Deardorff et al., 2013). *Peromyscus* mice are also established reservoirs for other pathogens such as *Borrelia* species responsible for Lyme disease and hantaviruses, which cause hantavirus cardio-pulmonary syndrome (Schmaljohn and Hjelle, 1997; Morzunov et al., 1998; Monroe et al., 1999; Barbour, 2016). However, infectious POWV/DTV has not been isolated from any wild-caught *Peromyscus* species to date. This may be because very few studies have attempted to isolate POWV from *Peromyscus* but have rather focused on serological surveys. Isolation of infectious virus from wild-caught *Peromyscus* would be indisputable evidence that these mice are a natural reservoir, but this has yet to be accomplished.

#### Experimental infection of *Peromyscus* mice

Based on the studies of (Deardorff et al., 2013), we developed an experimental model of POWV infection in *P. leucopus* (Mlera et al., 2017). Inoculation (by injection) of 4-week old *P. leucopus* mice with 10^3^ PFU of POWV (lineage I/LB strain) did not result in overt clinical signs of disease (Mlera et al., 2017). This observation was similar to results of a study in which POWV lineage II was used to subcutaneously infect adult *P. leucopus* mice (Telford et al., 1997). In our study, the lack of an apparent clinical disease was observed even when the mice were intracranially (ic) inoculated. However, mild signs of inflammation, such as perivascular cuffing and microgliosis were evident when brain sections were examined (Mlera et al., 2017). *In situ* hybridization also revealed that POWV was restricted to the olfactory bulb and ventricle in ic-inoculated *P. leucopus* mice. Analysis of the *P. leucopus* brain transcriptome following ic inoculation with POWV revealed that the mice responded by activation of the IFN-signaling system. *In vitro* experiments with *P. leucopus*-derived fibroblasts supported our observations that interferon is secreted in response to POWV (Izuogu et al., 2017). It remains undetermined, however, whether the IFN signaling pathway is the sole or most important system restricting POWV infection. We are in the process of data mining the genome of *P. leucopus* to gain further insight into the restriction of POWV in *P. leucopus*. Although the IFN secretion in *Myodes* cells does not eliminate virus replication (Stoltz et al., 2011), POWV is restricted in *P. leucopus* fibroblasts.

### TBFVs in *Apodemus* species

The *Apodemus* genus comprises more than 20 species (Bugarski-Stanojević et al., 2008). The center of origin of *A. agrarius* rodents is believed to be Eastern Russia, and this has dramatically expanded westward without human assistance (Aguilar et al., 2008; Hildebrand et al., 2013). The expansion is exemplified by the identification of *A. agrarius* in 59 new localities in south western Slovakia (Tulis et al., 2016). *A. flavicolis* breeding season begins in March and ends in October and females produce 2–3 litters each (Stenseth et al., 2002). Except for winters following heavy mast years, winter reproduction does not occur in *Apodemus* species (Adamczewska, 1961; Pucek et al., 1993).

The *A. agrarius*, species is the most abundant in Europe and Asia (Bugarski-Stanojević et al., 2008). A study investigating tick infestation of small mammals in an English woodland showed that *A. flavicolis* (giant yellow-necked mouse) was the most abundant (52.5% of 217), followed by *Apodemus sylvaticus* (wood mouse) at 35.5% (Cull et al., 2017). Unfortunately, this study did not look for evidence of virus infection either in the animals or in the ticks collected from the infested animals.

Several TBEV strains have been isolated from the *Apodemus* mouse species, indicating that the genus is a reservoir host for TBFVs. Examples include the TBEV strains KrM 93 and KrM 213, which were isolated from lung and spleen tissue harvested from *A. agrarius* (striped field mouse) caught in South Korea (Kim et al., 2008; Yun et al., 2011). In Hokkaido, Japan, the TBEV strains Oshima 08-As and Oshima-A-1 were isolated from spleens of wild-caught *A. speciosus* (large Japanese filed mouse). The studies reporting isolation of TBEV from *Apodemus* mice seem to suggest that organ tropism of TBEV in *Apodemus* mice is different from that in *Myodes* species. In other studies, TBEV RNA was also detected predominantly in the spleen and infrequently other organs, such as the brain, lung and blood clots (Knap et al., 2012). There was no TBEV RNA detected in the kidneys and liver (Knap et al., 2012). In addition, the viral loads in *Apodemus* mouse organs were generally lower (range 6.48–3.7 × 10^5^ copies/ml) when compared to those observed in *Myodes* species (Knap et al., 2012). Thus, it is apparent that TBFV organ tropism and extent of restriction varies between reservoir hosts.

Although the *Apodemus* mice seroconvert following exposure to TBFVs, the antibody titers reported in one study were several orders of magnitudes lower than in *Myodes* rodents. The titers determined by indirect immunofluorescence ranged from 0 to 80 in *A. flavivcolis* mice, compared to 0–1,280 in *Myodes* rodents (Knap et al., 2012). Furthermore, the study by Knap and colleagues showed lower seropositivity/infection rates in *Apodemus* than in *Myodes* rodents (Knap et al., 2012). They found that wild-caught *A. flavivcolis, A. sylvaticus*, and *A. agrarius* were seropositive for TBEV at 3.9, 9.6, and 2.4%, respectively (Knap et al., 2012). In a Hungarian study, 3.7% (3/327) *A. flavicolis* mice were positive for antibodies against TBEV whereas 4.6% (8/174) *A. agrarius* mice were seropositive for TBEV antibodies (Zöldi et al., 2015), further indication that the infection rate in *Apodemus* is relatively low.

The seropositivity and viral load differences between *Apodemus* and *Myodes* could be attributed to ecological factors. For example, a study looking at the effect of weather on the activity of the 2 rodent species showed that activity of *M. glareolus* rodents was positively influenced by moon phase regardless of cloud cover (Wróbel and Bogdziewicz, 2015). In contrast, rainfall positively impacted *A. flavicolis* but decreased activity of *M*. *glareolus* (Wróbel and Bogdziewicz, 2015). Thus, decreased rodent activity due to ecological factors could enhance infestation by ticks hence affecting seropositivity rates between species.

#### Experimental infection of *Apodemus* mice

Peroral, intraperitoneal or intramuscular inoculation of *Apodemus* mice results in no overt clinical signs of disease (Kopecky et al., 1991; Egyed et al., 2015). Wild-caught *A. sylvaticus* mice experimentally inoculated intraperitoneally with the virulent Central European (TBEV-Eur) strain were viremic for only 3 dpi (Kopecky et al., 1991). In *Apodemus* mice, TBEV was not detectable in the brains from 1 to 7 dpi (Kopecky et al., 1991), but, a recent study using only 2 wild-caught *A. agrarius* showed neuroinvasion and subclinical encephalitis following peroral inoculation (Egyed et al., 2015). Rodents that were intramuscularly inoculated showed no histological alterations in the brains, but mice that were orally inoculated with 1.5 × 10^3^ PFUs presented with viral antigens in the brain and this was accompanied by mild lympho-histiocytic vasculitis, which was restricted to the anterior olfactory nucleus (Egyed et al., 2015). The animal numbers used in this study (*n* = 2 per group) limit the conclusions that can be drawn from the study. The lack of neuroinvasion by TBEV in *Apodemus* mice (Kopecky et al., 1991) is similar to our observations when we inoculated *P. leucopus* mice via the peripheral route (Mlera et al., 2017).

In the studies by Kopecký and colleagues, the authors compared the response of *A. sylvaticus* mice to that of outbred ICR laboratory mice and reported that the macrophages from ICR mice had higher virus titers that were sustained for 7 dpi. In contrast, viral titers in *A. sylvaticus* macrophages rapidly declined from just above 10^4^ PFUs at day 0 to below 10 PFUs by 7 dpi (Kopecky et al., 1991). Macrophages are important cells in viral pathogenesis and they may function as virus reservoirs when infected by flaviviruses (Mogensen, 1979; Kreil and Eibl, 1995). Subversion of the anti-viral activities of macrophages by flaviviruses can facilitate viral replication and spread, enhancing the intensity of immune responses, leading to severe immune-mediated disease which may be further exacerbated during the subsequent infection with some flaviviruses (Ashhurst et al., 2013). Thus, the ICR mouse macrophages may be incapable of curbing TBEV infection, or the TBFVs may have evolved mechanisms to antagonize ICR mouse macrophage responses, but not those of *the Apodemus* species. This is in addition to the possibility that the *Apodemus* mice have evolved broader TBFV restriction mechanisms than ICR mice. Differences in the mechanistic responses of macrophages from various rodent species may be useful in understanding TBFV pathogenesis and could assist in the development of antiviral therapies.

IFN responses were mounted in both ICR and *A. sylvaticus* mice, but the serum levels peaked 1 dpi in ICR mice, the peak in *A. sylvaticus* mice was reached at 2 dpi. Notably, the highest IFN titers in *A. sylvaticus* mice were lower at 1,280, but the titer was extremely high at 10,240 in ICR mice. Thus, the overtly high IFN induction in ICR mice could lead to a runaway cytokine storm, perhaps leading to more aggravated pathogenesis in these mice.

Seroconversion appeared to be faster in the *A. sylvaticus* mice, detectable at 3 dpi and reaching titers of 256 at 7 dpi. In ICR mice, the titer reached 32 at 7 dpi. Although *A. sylvaticus* mice respond by an IFN and antibody response, it seems they are not able to clear TBFV infection completely, hence the detection of TBFV in wild-caught mice persists albeit at low level. Only mice inoculated with TBEV doses starting from 100 PFU seroconverted in the study reported by Egyed et al. (2015).

### The role of medium-sized mammals

The role played by medium-sized mammals (Figure 1) in the biology of TBFV infections remains poorly studied. Woodchucks (*Marmota monax*) are one such medium sized mammal that might play a role in TBFV ecology. The *I. cookei* ticks are believed to transmit mostly POWV Lineage I and these ticks preferentially infest woodchucks for blood meals (Ebel, 2010). Seroprevalence of POWV antibodies in adult wild-caught woodchucks in Ontario, Canada during the mid-summers of 1964 and 1966 was quite high in the range 43–60% (McLean et al., 1964, 1967). Juvenile woodchucks tested in 1964 had relatively lower seroprevalence rates in May (33%), but the rate increased dramatically to 47% by July (McLean et al., 1964). POWV was isolated from 3/60 pools of *I. cookei* ticks removed from these woodchucks caught in 1966, as well as from the blood of 2 animals sampled in 1964 (McLean et al., 1964, 1967). In a different study, a pool of 56 *I. cookei* nymphs collected in 1981 from a feral yearling woodchuck in Guelph, Ontario (Canada) was also positive for POWV (Artsob et al., 1984). Virus was also isolated from the blood obtained from the same woodchuck (Artsob et al., 1984), indicating that the animal was viremic and a possible source of virus for naïve ticks. However, it was not clear whether the viremia was due to an active recent infection or rather a persistent infection that had been acquired at an earlier time. Viremia was sustained for 8–11 days following experimental subcutaneous inoculation of woodchucks with POWV lineage I (Kokernot et al., 1969). Nevertheless, these reports demonstrate that woodchucks play an important role in the ecology of POWV in nature and might be useful as experimental models of infection to delineate specific host-pathogen interactions.

Studies conducted in Connecticut and Massachusetts showed that 16% (12/75) and 83% (10/12), respectively, of the striped skunk (*M. mephitis*) had hemagglutinin inhibiting antibodies against POWV (Main et al., 1979). Additional POWV seropositivity in skunks was reported for 1/5 of spotted skunks (*Spilogale putorius*) caught in Alameda County, California (Johnson, 1987), however, none of the 4 striped skunks caught in the same study tested positive. There is a single isolation of POWV from the kidneys of an apparently healthy male spotted skunk in California (Johnson, 1987). However, efforts to isolate virus from the brain, trachea, lungs or throat swabs from the same animal failed (Johnson, 1987). These results suggest that POWV may persist in the kidneys of spotted skunks, which is in contrast to the brain as in the *Apodemus* or *Myodes* species. Considered together, these results suggest that the skunk is a true reservoir of POWV in nature, but additional studies are required to evaluate the current seropositivity status and/or isolation of virus from tissues obtained from these animals.

The raccoon (*Procyon lotor*) is another medium sized mammal that can be infested by TBFV-transmitting *Ixodes* and *Hemaphysalis* ticks (Jinnai et al., 2009; Inoue et al., 2011). Thirty three raccoons captured in New York State harbored POWV/DTV-infected ticks, but none of the animals were seropositive for POWV (Dupuis et al., 2013). Inoculation of captured raccoons with POWV lineage I did not result in viremia or clinical signs of disease (Kokernot et al., 1969). Apart from these reports, no other study that we are aware of describes POWV biology in the raccoon. Thus, additional studies are required to elucidate the relationship between this ubiquitous animal and TBFVs.

Apart from the isolation of the WV77 DTV in West Virginia (USA) from a fatal case of encephalitis in the gray fox, *Urocyon cinereoargenteus* (Kuno et al., 2001), no field reports have described seroprevalence. However, experimental inoculation of this member of the Canidae family with a very large dose of 540,000 LD_50_ POWV (lineage I) resulted in no obvious clinical signs of disease although viremia was observed for 3 dpi (Kokernot et al., 1969). In addition, no signs of clinical disease were observed following subcutaneous challenge of the red fox (*Vulpes vulpes*) (Kokernot et al., 1969). This is interesting considering that the WV77 caused a fatality, and it begs the question as to what extent experimental inoculation can mimic natural infection via tick bite. Perhaps, the animal might have had other underlying conditions or a mixed infection, which aggravated the DTV infection.

Companion animals in the Canidae family, such as dogs, are also prone to tick infestations that could lead to transmission of TBFVs (Bajer et al., 2013; Chen et al., 2014). Clinical TBEV infection in dogs is rarely diagnosed, and is likely to be fatal in most cases (Pfeffer and Dobler, 2011). Fatality is preceded by fever, aggressiveness, optic neuritis, and encephalitis (Stadtbaumer et al., 2004; Pfeffer and Dobler, 2011; Bajer et al., 2013). Surveillance studies done in Austria, Belgium, the Czech Republic, Denmark, Germany, Japan, Norway and Sweden showed seropositivity in dogs ranging from 0.1 to 24.1% (reviewed in Pfeffer and Dobler, 2011). The studies from Germany and the Czech Republic indicated that there were some neurological symptoms observed in the sero-surveillance, but the clinical outcomes were not specified (Pfeffer and Dobler, 2011). Thus, TBFV infection of canines requires further study.

### Cervids and other large mammals

Large mammals, such as deer and elk (Figure 1) are inadvertent and transient TBFV hosts, which play an important role predominantly in maintenance of tick populations by providing blood meals (Carpi et al., 2008). Non-viremic transmission from infected ticks to naïve ticks cofeeding on the same host in proximity is well documented and the large animals also contribute to this process. From 1979 to 2010, 32% (84/266) of white-tail deer serum samples collected from Connecticut were positive for antibodies against POWV/DTV (Nofchissey et al., 2013). A recent survey also found evidence of antibodies against POWV in 4.2% of Eastern US white tail deer, indicating that virus-infected ticks continue to feed on this large mammal (Pedersen et al., 2017). Seropositivity in these large animals is a useful sentinel marker of TBFV prevalence in geographic regions in which the animals are found (Tonteri et al., 2016). It is evident from seropositivity that the cervids use the adaptive immune response to ward off TBFV infection, but specific innate and molecular responses need to be elucidated further especially since there is no evidence of neuroinvasion/neuropathology.

In some rural parts of Europe, goats and sheep are reared for milk. The milk may be consumed raw or as processed milk products, such as cheese. Recent reports have shown that sheep may develop encephalitis after natural TBEV infection (Böhm et al., 2017) Infected milk goats infected with TBEV can shed the virus in the milk and the virus can be transmitted to humans who consume it unpasteurized (Ernek et al., 1968; Cisak et al., 2010; Balogh et al., 2012; Hudopisk et al., 2013; Offerdahl et al., 2016). Goats have also been proposed as sentinels for TBEV in endemic areas (Klaus et al., 2012; Rieille et al., 2017; Salat et al., 2017). Experimental infection of goats with TBEV leads to no clinical signs of disease even when virus is being shed in the milk (Balogh et al., 2012). This is interesting, and it would be interesting to know which cells of the animals' mammary apparatus harbor the virus.

## Future prospects

In this paper, we reviewed the role of small-to-medium-sized mammals in TBFV biology. Rodents, particularly the vole (*Myodes*) and yellow-necked mouse (*Apodemus*), are true reservoirs of the viruses because viruses have been isolated from animals without clinical signs of disease. These are probably the most important rodent reservoirs of TBFVs. Although the role of the rodents is indisputable, very little research has been done to evaluate the specific host-pathogen interactions in these animals. The dearth of knowledge extends to medium-sized mammals, although some early reports indicate that woodchucks and skunks are critical players in the ecology and biology of TBFVs.

Studies designed to understand the role of reservoirs species will be important to develop the complete natural history of TBFV. Our lab is actively pursuing *in vivo* experiments as well as with cell cultures derived from some of these animals as surrogates for understanding permissiveness to infection as well as elucidating host cell factors which are critical for either susceptibility or resistance to TBFV infection.

## Author contributions

All authors listed have made a substantial, direct and intellectual contribution to the work, and approved it for publication.

### Conflict of interest statement

The authors declare that the research was conducted in the absence of any commercial or financial relationships that could be construed as a potential conflict of interest.
